# Personality Traits and Weight Loss Maintenance: A Cross-Sectional Study

**DOI:** 10.3389/fnut.2021.702382

**Published:** 2021-07-01

**Authors:** Yiannis Koutras, Stavri Chrysostomou, Konstantinos Giannakou, Mary H. Kosmidis, Mary Yannakoulia

**Affiliations:** ^1^Department of Life Sciences, School of Sciences, European University Cyprus, Nicosia, Cyprus; ^2^Department of Health Sciences, School of Sciences, European University Cyprus, Nicosia, Cyprus; ^3^Laboratory of Cognitive Neuroscience, School of Psychology, Aristotle University of Thessaloniki, Thessaloniki, Greece; ^4^Department of Nutrition and Dietetics, School of Health Sciences and Education, Harokopio University, Athens, Greece

**Keywords:** obesity, weight loss maintenance, personality, impulsiveness, diet

## Abstract

**Purpose:** This cross-sectional study was based on the Cypriot cohort of the MedWeight study and examined differences between maintainers and regainers regarding personality traits.

**Methods:** Participants were men and women who reported being at least overweight and experienced an intentional weight loss of ≥10% of their maximum weight, at least 1 year before participation. Assessment of personality, diet and physical activity was conducted through validated questionnaires and with 24 h recalls.

**Results:** Findings from logistic regression analysis indicated that the odds of maintaining weight loss increased to 50% for agreeableness and decreased to 20 and 7% for perseverance and motor impulsiveness, respectively.

**Conclusion:** Specific aspects of personality and impulsivity are relevant to weight loss maintenance and need to be considered when developing weight management interventions.

## Introduction

In recent years, there has been a growing research interest in the study of obesity and personality traits to enhance our understanding of personality traits associated with body weight, overweight and obesity, as well as the effectiveness of weight loss interventions (1–3). In particular, “neuroticism”, “impulsivity,” and “sensitivity to reward” have been shown to be risk factors, whereas “conscientiousness” and “self-control” have been shown to have a protective function in relation to weight gain (2, 3). Moreover, low “agreeableness” and impulsivity-related traits have been found to predict a greater increase in Body Mass Index (BMI) across the adult life span, whereas high “neuroticism” and low “conscientiousness” have been associated with long term weight fluctuations (3).

Regarding the post-dieting period, identification of specific personality traits associated with weight loss maintenance may enhance our knowledge of psychological aspects of obesity and the development of increasingly effective weight loss maintenance-intervention programs. However, the data are limited. Among English language papers, so far only one study, specifically, the Finnish Weight Control Registry, has indicated that personality traits, such as neuroticism, agreeableness, and conscientiousness, may be important in successful long-term weight loss maintenance (4). The aim of this study was to explore differences in personality traits and personality dimensions of impulsiveness, between maintainers and regainers enrolled in the MedWeight control registry in Cyprus.

## Materials and Methods

### Study Population

The current cross-sectional study included 232 participants from the MedWeight, a registry of people who lost weight and maintained it (maintainers) or regained the loss (regainers) (5). Inclusion criteria were men and women aged 18–65 years of Cypriot ethnicity without any mental disorder, who reported being at least overweight (BMI ≥25 kg/m^2^) and experienced an intentional weight loss of ≥10% of their maximum weight at least 1 year before participation in the study. Each eligible participant was classified as “maintainer” if his/her current weight was ≤90% of his/her maximum weight or “regainer” if his/her current weight was ≥95% of his/her maximum weight. To avoid overlapping of groups, participants who had a current weight between 91 and 94% of their maximum weight were excluded. Current pregnancy was also an exclusion criterion.

This study was conducted according to the Declaration of Helsinki guidelines. All procedures involving research study participants were approved by the Cyprus National Bioethics Committee (EEBK.∏.2016.01.29) and all eligible participants signed a Consent Form before study participation. The recruitment procedure lasted 2 years (12/2017–12/2019) and occurred through media (i.e., television, radio, press releases). Upon entry, eligible volunteers were advised to access a website platform (http://medweight.hua.gr) to fill in a series of questionnaires regarding socio-demographic status such as marital status (single, married/cohabitating, divorced, widowed, then coded for married/cohabitating or not), occupational status (employed or not) and years of education. Participants were asked to report physical/personal characteristics such as sex, age, weight, height, BMI, maximum weight, maximum BMI, initial loss, maintenance loss, and duration of maintenance.

### Assessment of Dietary Intake

Dietary intake was evaluated using two 24-h dietary recalls for each participant, 10 days apart from each other, with weekdays and weekends proportionately represented among participants (6, 7). The recalls were performed by well-trained dietitians using the multiple-pass method (8, 9). It is noted that dietitians were blinded as to whether the participant was a maintainer or regainer. Volunteers were asked to report in detail all foods and beverages consumed on the day before (i.e., between waking up in the morning and going to bed at night). For each eating occasion, information regarding time, location, parallel activities, and companions were also recorded. In addition, participants were not informed when the recalls would be occurred, ensuring no modifications in their diet in anticipation of the recall. All data were analyzed in terms of total daily energy intake by using the dietary analysis software SNPRO Nutrition Software (Cheapsoft Softwares, 2017).

### Assessment of Physical Activity

The short version of the International Physical Activity Questionnaire (IPAQ) validated for the Greek population was used to assess physical activity (10). Participants were asked to answer 7 questions in order to record the number of days (frequency) and the number of minutes per day (duration) of their participation in all kinds of vigorous, moderate and walking activities lasting ≥10 min during the last seven days. In addition, participants were asked about the time spent sitting during an average weekday.

### Assessment of Personality

The Ten-Item Personality Inventory (TIPI) was used to assess participants' personality (11). It is a 10-item survey based on 5 personality dimensions (conscientiousness, openness, neuroticism, agreeableness, and extraversion) that measures the essential characteristics of an individual's personality using a 7-point scale (1 = strongly disagree to 7 = strongly agree). For the purposes of the study, TIPI was translated into Greek and then reversely translated was held to check the reliability of the translation, which was found to be satisfactory after two-point conversion.

The Greek version of the Barratt Impulsiveness Scale (BIS) was used to assess personality dimensions of impulsiveness (12, 13). It is a 30-item self-report questionnaire scored on a 4-point scale (1 = never/rarely to 4 = almost always/continuously) to yield six first-order factors (attention, motor, self-control, cognitive complexity, perseverance, cognitive instability) and three second-order factors (attentional, motor, non-planning impulsiveness). Total score ranges from 30 to 120, with high score indicating high impulsiveness.

### Statistical Analysis

Descriptive statistics were used to summarize the baseline demographic characteristics of the study participants. Normally distributed numeric variables were presented as means and standard deviation (SD) and as medians and interquartile range (IQR) for numeric measures with skewed distributions. Data from categorical variables were presented as absolute (*n*) and relative (%) frequencies. Normality of distribution of data was explored by visual inspection of Q-Q plots. We explored differences between maintenance status in participants' characteristics using independent *t*-test or Mann Whitney rank tests, depending on the normality of the data, and chi-square tests for categorical variables.

Logistic regression analysis was used to explore differences between maintainers and regainers, using maintenance status as a dependent variable (1 = maintainer, 0 = regainer) for categorical variables and the results were expressed as Odds Ratio (OR) and 95% confidence interval (CI). Logistic regression model 1 was adjusted for age, sex, and marital status (married or not); model 2 was additionally adjusted for energy intake and model 3 was additionally adjusted for physical activity (IPAQ total Met-minutes per week).

We also conducted a series of mediational analyses with dichotomous outcome (14), to determine whether initial weight loss mediated the association between agreeableness or attentional impulsiveness or motor impulsiveness or total BIS score and maintenance status. Mediation analyses were conducted using procedure previously described (15). Using the SPSS macro provided by Preacher and Hayes (16), we calculated total, direct and indirect effects, including tests of significance using bootstrap procedures. All reported *p*-values were 2-tailed, and α = 0.05 was used as to determine statistical significance. Statistical analysis was performed using SPSS Statistics 22.0.

## Results

Two hundred and thirty-two participants enrolled in this study, 145 maintainers and 87 regainers. Both men and women were equally represented in the total sample, as well as within the maintainers and regainers groups (*p* = 0.893) (Table 1). As a group, maintainers were younger and more educated compared with regainers. Current weight and BMI of maintainers were lower, but their maximum weight, maximum BMI ever reached as well as their initial weight loss was significantly greater compared with regainers (*p* < 0.05 for all). A mean weight loss of 12.5% for almost 3.5 years was reported in the maintainers group. Regarding physical activity, maintainers reported significantly higher physical activity level than regainers (*p* < 0.001).

**Table 1:** Participants' characteristics.

	**Total (*n* = 232)**	**Maintainers (*n* = 145)**	**Regainers (*n* = 87)**	***P*-value**
Sex (% female)	52.20%	51.70%	52.90%	0.893
Marital status (% married)	29.70%	33.10%	41.40%	0.396
Employment status (% employed)	60.20%	69.40%	72.90%	0.524
Age (years)	34.61 ± 13.21	33.00 ± 12.19	37.29 ± 14.43	**0.022**
Weight (kg)	82.41 ± 18.86	77.61 ± 17.49	90.43 ± 18.43	**0.001**
BMI (kg/m^2^)	28.67 ± 5.48	26.90 ± 4.86	31.60 ± 5.19	**0.001**
Max weight (kg)	96.13 ± 22.10	98.38 ± 23.61	92.38 ± 18.83	**0.045**
Max BMI (kg/m^2^)	33.41 ± 6.13	34.08 ± 6.45	32.29 ± 5.40	**0.031**
Initial loss (kg)	22.46 ± 14.52	26.72 ± 15.96	15.37 ± 7.62	**0.001**
Weight loss maintained (%)		12.44 ± 12.66		
Maintaining years		3.37 ± 3.14		
Education years	14.45 ± 3.69	15.11 ± 3.18	13.31 ± 4.20	**0.002**
Physical activity (MET*min/week)	2409.54 ± 2737.63	2228.5 ± 3062	720 ± 1384	**0.001**

*Values are presented as mean ± standard deviation for quantitative variables and relative frequencies for qualitative variables, BMI: Body mass index, statistically significant group differences are denoted in bold*.

Table 2 reports the comparisons of the two groups on the five personality dimensions of the TIPI questionnaire and the BIS scale. The only statistical difference regarding the five personality dimensions was observed for agreeableness. Specifically, maintainers scored higher on agreeableness compared with regainers (*p* = 0.009). Regarding the results of the BIS, maintainers scored significantly lower on attention (*p* = 0.012) and attentional impulsiveness (*p* = 0.016) compared with regainers. A significant difference between the two groups (*p* < 0.05) was also observed on perseverance and motor impulsiveness, as well as on the total BIS score, with higher scores in the regainers relative to the maintainers (Table 2).

**Table 2:** Comparison of personality traits between maintainers and regainers.

**Dimensions of TIPI**	**Maintainers (*n* = 109)**	**Regainers (*n* = 64)**	***p*-value**
Extraversion	4.59 (1.289)	4.38 (1.458)	0.321
Agreeableness	5.413 (1.1239)	4.953 (1.0605)	**0.009**
Conscientiousness	5.362 (1.1684)	5.164 (1.3125)	0.305
Emotional stability	4.211 (1.1947)	4.180 (1.2952)	0.872
Openness to experiences	5.11 (1.121)	4.95 (1.302)	0.390
**Dimensions of BIS**			
Attention	9.64 (2.251)	10.58 (2.455)	**0.012**
Cognitive instability	6.61 (1.948)	7.02 (1.906)	0.180
Attentional impulsiveness	16.25 (3.394)	17.59 (3.715)	**0.016**
Motor	13.75 (3.786)	14.83 (4.614)	0.098
Perseverance	6.74 (1.740)	7.98 (2.440)	**0.001**
Motor impulsiveness	20.5 (21.067)	22.81 (5.955)	**0.009**
Self-control	12.65 (3.345)	13.30 (3.379)	0.224
Cognitive complexity	13.17 (2.968)	13.17 (2.640)	0.996
Non-planning impulsiveness	25.83 (5.171)	26.47 (4.567)	0.411
Total BIS score	62.57 (10.901)	66.88 (11.168)	**0.014**

*Values are presented as mean ± standard deviation. Statistically significant group differences are denoted in bold*.

We have also performed a logistic regression analysis using three different models. After adjusting for marital status, age and sex, agreeableness was positively associated with weight loss maintenance (Model 1: *p* < 0.05, OR 1.59, 95% CI: 1.17–2.18) and remained statistically significant across all models (Model 2: *p* < 0.05, OR 1.60, 95% CI: 1.17–2.19) and (Model 3: *p* < 0.05, OR 1.47, 95% CI: 1.07–2.04). In addition, after adjustment for marital status, age and sex (Model 1), attention (*p* < 0.05, OR 0.82, 95% CI: 0.71–0.95), attentional impulsiveness (*p* < 0.05, OR 0.88, 95% CI: 0.80–0.97), motor (*p* < 0.05, OR 0.92, 95% CI: 0.84–0.99), perseverance (*p* < 0.001, OR 0.75, 95% CI: 0.63–0.88), motor impulsiveness (*p* < 0.05, OR 0.90, 95% CI: 0.84–0.96), as well as total BIS score (*p* < 0.05, OR 0.96, 95% CI: 0.93–0.99) were inversely associated with weight loss maintenance. Moreover, after additionally adjusting for energy intake (Model 2), results remained statistically significant across the dimensions of the BIS scale. However, after further adjustment for physical activity (Model 3), only perseverance (*p* < 0.05, OR 0.81, 95% CI: 0.67–0.96) and motor impulsiveness (*p* < 0.05, OR 0.93, 95% CI: 0.86–0.99) remained statistically significant.

In regards to the results from mediation analyses, initial weight loss did not significantly mediate the association between agreeableness [Indirect Effect (IE) = 0.101, 95% CI −0.289, 0.460] or any of the dimensions of impulsiveness (attentional: IE = 0.04, 95% CI −0.045, 0.155 and motor: IE = −0.009, 95% CI −0.060, 0.039) or total BIS score (IE = 0.001, 95% CI −0.027, 0.028) and weight loss maintenance.

## Discussion

The present cross-sectional study examined the association between personality traits and weight loss maintenance among adults who had previously lost weight and either maintained it or regained it. Our results indicate that maintaining weight loss is directly associated with agreeableness, whereas impulsiveness and specific dimensions of impulsiveness (attentional and motor impulsiveness) are inversely associated with weight loss maintenance. Hence, specific aspects of personality and impulsivity are relevant to weight loss maintenance and should be considered when developing weight management interventions.

Previous research regarding individuals with obesity have yielded lower scores in this group relative to their normal weight peers with respect to personality traits such as agreeableness (17). Therefore, it is not surprising that our results suggest a protective role of agreeableness in weight loss maintenance, perhaps through a mechanism involving lower susceptibility to hunger (18). What was also evident and in line with previous results (19), was that the protective role of agreeableness remained statistically significant even after adjusting for several confounding factors, indicating that the relationship between agreeableness and weight loss maintenance is probably not moderated by factors such as age, sex, marital status, energy intake or even physical activity. Even though maintainers and regainers differed in terms of reported initial weight loss, the results from mediation analysis revealed that the relationship between agreeableness and weight loss maintenance was not mediated by factors such as initial weight loss, thus it failed to explain the relationship in more detail.

Of interest, the present findings are also in line with previous findings in relation to impulsiveness (20). A previous cross-sectional study of 11,929 men and 39,114 women, showed that highly impulsive participants were more likely to report a BMI > 40 kg/m^2^ and supports the importance of psychological factors in the prevention of obesity. Moreover, specific dimensions of impulsivity, such as “attentional” and “motor” have been positively associated with increased BMI and binge eating (21). This was also reflected in our results, where it was evident that “attentional” and “motor” impulsiveness, as well as the total impulsivity score were associated with lower odds of weight loss maintenance. However, this association was not mediated by initial weight loss as was hypothesized at the beginning. One explanation may be that when both “attentional” and “motor” impulsivity levels are elevated, as was evident in the regainers group, control over self-eating tends to weaken (22, 23).

It is critical to keep in mind that successful weight maintenance is associated not only with personality traits, but also with other factors such as specific eating behaviors (24) and behaviors related with physical activity (25). Our results revealed a higher level of physical activity reported by maintainers compared to regainers. Previous research highlighted the important role of physical activity in weight loss maintenance (26). Activity patterns may differ between men and women, however, adherence to a high-level physical activity pattern is considered critical for long term weight loss success (26–28). In the present study, data regarding physical activity were used in regression models and highlighted that statistically significance threshold was remained significant even after adjusting for physical activity.

Additionally, age is considered as a non-modifiable factor more likely affecting the effort of weight loss maintenance. The NHANES study with over 14,000 participants indicated that weight loss maintenance was achieved among those who were older compared to younger participants (ages 75–85 vs. 20–35) (29). Most recently, a randomized control trial among adults with overweight/obesity reported that participants of age ≥60 years had greater initial weight loss and greater sustained weight loss compared to younger adults (30). On the contrary, in our study we found that maintainers were younger than regainers. Notably, the mean age for maintainers and regainers of our study was under 40 years. Therefore, it could be assumed that age is more likely to affect long-term weight loss success among older adults, over 60 years.

The present study has limitations. Although involving maintainers and regainers in our sample allowed for direct comparisons of these two groups, yet, the observational design of the study does not imply causality, but merely associations. In addition, physical activity was evaluated by a self-report questionnaire, which could lead to misreporting and information bias. However, IPAQ is a widely used tool with high reliability and validity (10, 31).

## Conclusion

Specific aspects of personality and impulsivity appear to play a key role in weight loss maintenance. Findings must be taken with caution because of the cross-sectional nature of the study that does not allow to draw conclusions about etiological connections. Future research should examine if personality traits through their association with other psychological factors, could influence weight loss maintenance and whether these parameters need to be considered in personalizing interventions in the dieting and post-dieting periods.

## Data Availability Statement

The raw data supporting the conclusions of this article will be made available by the authors, without undue reservation.

## Ethics Statement

The studies involving human participants were reviewed and approved by Cypriot National Bioethics Committee (EEBK.∏.2016.01.29). The patients/participants provided their written informed consent to participate in this study.

## Author Contributions

YK: conceptualization, methodology, formal analysis, investigation, data curation, and writing—original draft. SC: validation, investigation, resources, writing—review and editing, and supervision. KG: methodology, validation, and writing—review and editing. MK: conceptualization, methodology, and writing—review and editing. MY: conceptualization, methodology, writing—review and editing, visualization, and project administration. All authors contributed to the article and approved the submitted version.

## Conflict of Interest

The authors declare that the research was conducted in the absence of any commercial or financial relationships that could be construed as a potential conflict of interest.
